# The lower respiratory tract microbiome of critically ill patients with COVID-19

**DOI:** 10.1038/s41598-021-89516-6

**Published:** 2021-05-12

**Authors:** Paolo Gaibani, Elisa Viciani, Michele Bartoletti, Russell E. Lewis, Tommaso Tonetti, Donatella Lombardo, Andrea Castagnetti, Federica Bovo, Clara Solera Horna, Marco Ranieri, Pierluigi Viale, Maria Carla Re, Simone Ambretti

**Affiliations:** 1 Operative Unit of Clinical Microbiology, IRCCS Azienda Ospedaliero-Universitaria di Bologna, 9 via G. Massarenti, 40138 Bologna, Italy; 2 Wellmicro s.r.l, Via Piero Gobetti, 101, 40129 Bologna, Italy; 3 Alma Mater Studiorum – Università di Bologna, Dipartimento di Scienze Mediche e Chirurgiche, Operative Unit of Infectious Diseases, IRCCS Azienda Ospedaliero-Universitaria di Bologna, Bologna, Italy; 4 Alma Mater Studiorum – Università di Bologna, Dipartimento di Scienze Mediche e Chirurgiche, Anesthesia and Intensive Care Medicine, IRCCS Azienda Ospedaliero-Universitaria di Bologna, Bologna, Italy

**Keywords:** Clinical microbiology, Clinical microbiology

## Abstract

COVID-19 infection may predispose to secondary bacterial infection which is associated with poor clinical outcome especially among critically ill patients. We aimed to characterize the lower respiratory tract bacterial microbiome of COVID-19 critically ill patients in comparison to COVID-19-negative patients. We performed a 16S rRNA profiling on bronchoalveolar lavage (BAL) samples collected between April and May 2020 from 24 COVID-19 critically ill subjects and 24 patients with non-COVID-19 pneumonia. Lung microbiome of critically ill patients with COVID-19 was characterized by a different bacterial diversity (PERMANOVA on weighted and unweighted UniFrac Pr(> F) = 0.001) compared to COVID-19-negative patients with pneumonia. *Pseudomonas alcaligenes*,* Clostridium hiranonis*,* Acinetobacter schindleri*, *Sphingobacterium* spp.,* Acinetobacter* spp. and *Enterobacteriaceae*, characterized lung microbiome of COVID-19 critically ill patients (LDA score > 2), while COVID-19-negative patients showed a higher abundance of lung commensal bacteria (*Haemophilus influenzae*, *Veillonella dispar*, *Granulicatella* spp., *Porphyromonas* spp., and *Streptococcus* spp.). The incidence rate (IR) of infections during COVID-19 pandemic showed a significant increase of carbapenem-resistant *Acinetobacter baumannii* (CR-Ab) infection. In conclusion, SARS-CoV-2 infection and antibiotic pressure may predispose critically ill patients to bacterial superinfection due to opportunistic multidrug resistant pathogens.

## Introduction

Coronavirus disease 2019 (COVID-19), caused by the severe acute respiratory syndrome coronavirus 2 (SARS-Cov-2), has spread worldwide causing more than 11,500,000 cases and 500,000 deaths. Since March 2020, the WHO has declared the global pandemic of COVID-19 a public health emergency^1^. Clinical symptoms of COVID-19 infections range from mild or moderate flu-like symptoms to severe pneumonia requiring oxygen support. In certain cases, COVID-19 disease may further progress to respiratory failure, acute respiratory distress syndrome (ARDS) and multiorgan failure^2^.

Viral respiratory infections (i.e. influenza A and B, respiratory syncytial virus, rhinovirus, human coronavirus) may predispose patients to secondary bacterial and/or fungal co-infections associated with high mortality rates^3^. In contrast to other betacoronaviruses (i.e. SARS-CoV-1 and MERS-Co-V), SARS-CoV-2 has been associated with an increase of secondary bacterial and/or fungal infections^4^. Previous studies showed that bacterial or fungal co-infections in COVID-19 patients are associated with poor clinical outcome, especially in critically ill patients^5,6^. Recently, Fan et al.^7^ observed that lung microbiota of deceased patients with COVID-19 exhibited complex bacterial and fungal colonization by opportunistic species. Yet despite the increasing number of COVID-19 studies, the predisposition of COVID-19 patients to secondary infection is not fully understood.

The aim of this study is to investigate the bacterial composition of lower respiratory tract from patients with SARS-CoV-2 infection and examine the association of opportunistic infection with gram-negative pathogens in association with their relative abundance in the lung microbiome.

## Results

### Clinical characteristics

The lower respiratory microbiome was characterized in 24 critically ill patients with COVID-19 and in 24 non-infected patients. The median (IQR) age was 68 (59–62) for the COVID-19-positive patients and 64 (50–71) for the SARS-CoV-2-negative patients; COVID-19-positive subjects were 29% female and 71% male (n = 24). COVID-19-negative patients were 42% female and 58% male (n = 24). Clinical characteristics of patients with COVID-19 and negative patients are summarized in Table 1.

**Table 1:** Clinical characteristics of critically ill patients with COVID-19 compared with negative subjects with pneumonia.

	Patients with COVID-19 N = 24 (%)	Patients with pneumonia N = 24 (%)	*p* value
**Demographics**			
Age, median (IQR)	68 (62–59)	64 (50–71)	0.45
Gender (Male)	17 (71)	14 (61)	0.47
Charlson comorbidity index	4 (4–4)	4 (3–5)	0.78
**Hospitalization**			
Length-of in-hospital stay, median (IQR) days	37 (28–55)	19 (6–30)	< 0.001
Time from hospital admission to respiratory sample, median (IQR) days	25 (12–27)	15 (5–17)	0.15
Time from ICU admission to respiratory sample, median (IQR) days	0 (0–3)	0 (0–5)	0.34
Time from COVID-19 diagnosis to respiratory sample, median (IQR) days	10 (1–16)	4 (1–8)	< 0.005
Time from symptoms onset to respiratory sample, median (IQR) days	18 (11–27)	15 (5–20)	0.14
Time from antimicrobial treatment to respiratory sample, median (IQR) days	2 (0–10)	8 (3–19)	0.45
ICU admission	23 (96)	3 (13)	< 0.001
Mechanical ventilation	24 (100)	6 (25)	< 0.001
Days of mechanical ventilation, median (IQR)	16 (13–23)	3 (2–15)	< 0.001
Time from intubation to respiratory sample, median (IQR) days	9 (3–19)	1 (0–13)	0.04
Total BAL samples (BAL per patients)	118 (5)	51 (2)	
**Radiological findings**			
Interstitial pneumonia	24 (100)	9 (37)	< 0.001
Multifocal	0	8 (33)	
Single infiltrate/Nodules	0	5 (21)	
Cavitating pnuomonia	0	2 (8)	
**Treatments**			
Hydroxycholoroquine	24 (100)	1 (4)	< 0.001
*Antivirals*			
Darunavir	3 (12)	0 (0)	< 0.001
Remdesivir	1 (4)	0 (0)	
Lopinavir/ritonavir	10 (42)	0 (0)	
INF	5 (21)	0 (0)	< 0.001
Tocilizumab	13 (54)	0 (0)	< 0.001
Corticosteroids	10 (42)	3 (13)	0.049
Antibiotics	13 (54)	14 (60)	0.88
BL/BLI	8 (33)	4 (17)	0.31
Cephalosporin	2 (8)	6 (26)	0.13
Carbapenem	3 (12)	2 (9)	0.99
Other	7 (29)	6 (26)	0.83
In-hospital mortality	8 (33)	1 (4)	< 0.001

All COVID19-positive patients were in Intensive Care Unit (ICU) on mechanical ventilation and received treatment with hydroxychloroquine. In addition, 10 patients (42%) received corticosteroids, 14 (59%) received antiviral therapy, 18 (75%) received immunomodulators (n = 18, 75%) and 16 (67%) received antibiotics in combination with hydroxychloroquine. The comparison group included 24 patients admitted to hospital presenting clinical and radiological findings of pneumonia and with PCR performed on nasopharyngeal swab or BAL negative for SARS-CoV-2. Radiological findings of the COVID-19-negative group were consistent with interstitial pneumonia in 9 (37%) patients, or patchy multifocal infiltrates in 8 (33%) cases, or other radiological findings in the remaining cases (7, 29%) as reported in Table 1. Compared with COVID-19 negative cohort, COVID-19 patients had longer in-hospital stay (37 vs. 19 days, *p* < 0.001) and in-hospital mortality (33% vs. 4%, *p* < 0.001).

### Lung microbiome dysbiosis in critically ill patients with COVID-19

**Figure 1:**
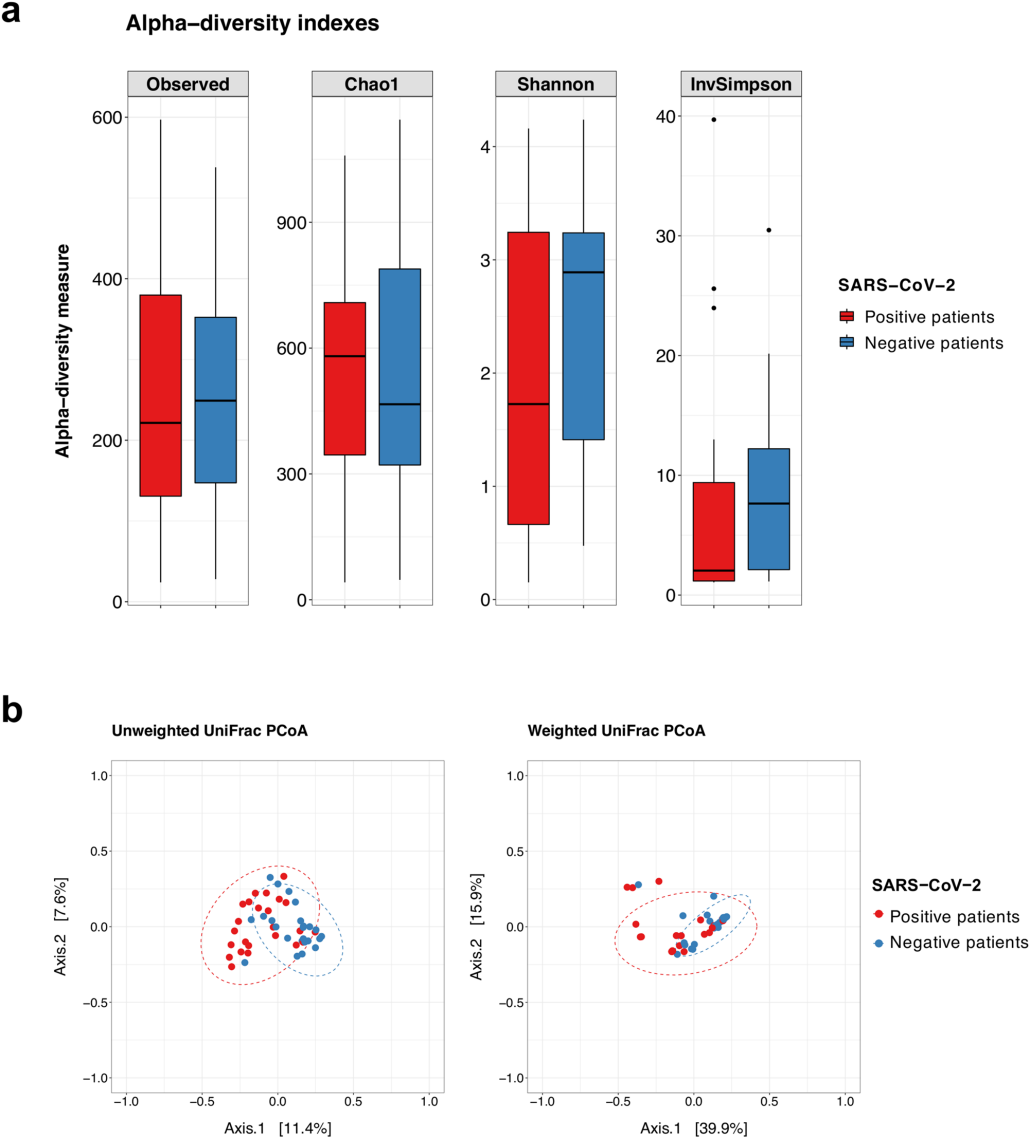
(**a**) Boxplots with whiskers showing the comparison of alpha diversity measures between SARS-CoV-2 positive patients (n = 24) and negative patients (n = 24). No significant differences were found between the two study groups. Median, first and third quartile and outliers are shown. (**b**) Principal Coordinate Analysis (PCoA) on unweighted and weighted UniFrac distance metric at the OTU level calculated on COVID-19 positive (n = 24, red dots) and COVID-19 negative patients (n = 24, blue dots). Each sample is represented by a dot. Axis 1 explained 12% and 40% of the variation observed, in the left and right graph, respectively, and Axis 2 explained 8% and 16% of the variation, in the left and right graph, respectively.

**Figure 2:**
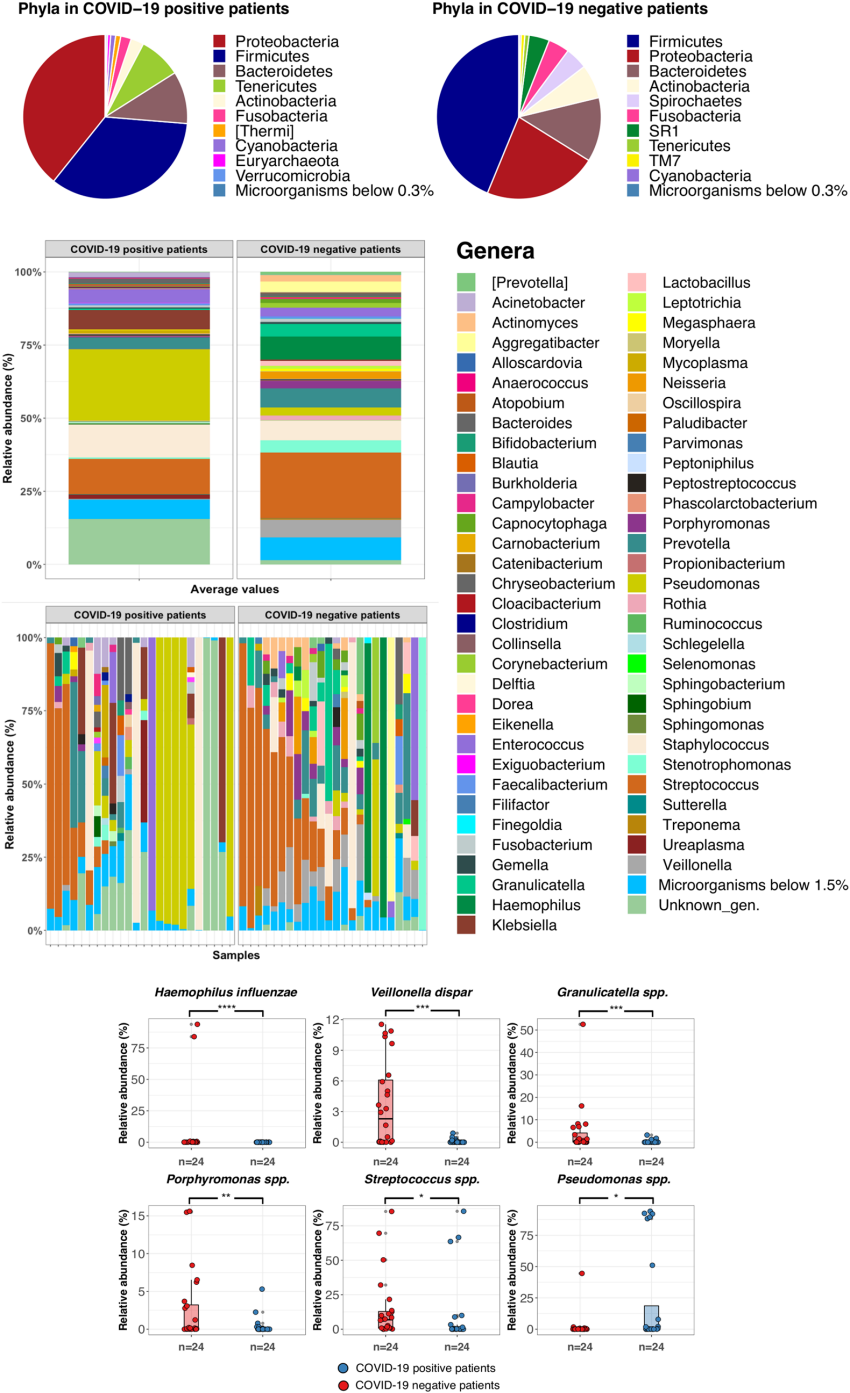
Taxonomic profiles at the phylum (pie charts) and genus level (stacked barplot) of COVID-19 critically ill patients and negative subjects. Taxa that were present in significantly different relative abundances after SIMPER analysis are shown in the lower panel.

Analysis of lung microbiome composition of COVID-19 critically ill patients revealed no significant differences in alpha diversity in comparison to COVID-19 negative patients (Fig. 1a). To assess the presence of compositional modifications in the two microbial communities, the Permutational Multivariate Analysis of Variance (PERMANOVA) on weighted and unweighted UniFrac was performed. The pulmonary microbiota of COVID-19 positive patients showed a significant difference of the centroids of the clusters versus COVID-19-negative patient microbiomes for weighted and for unweighted analysis (*p* value [Pr(> F)] = 0.001, with homogeneous and non-homogeneous dispersion of data [Pr(> F)] = 0.02, respectively) as shown in Fig. 1b. These findings indicate that compositional differences were associated with the presence or absence of specific taxa and their relative abundances in the samples. The lung microbiota profile of critically ill patients with COVID-19 was dominated by phyla Proteobacteria (48%), Firmicutes (37%), and Bacteroidetes (9%). Dominant bacterial families were *Pseudomonadaceae* (25%), *Enterobacteriaceae* (19%), *Streptococcaceae* (12%), *Staphylococcaceae* (11%). At the same time, Pseudomonas (25%), *Streptococcus* (12%), an unknown genus of the *Enterobacteriaceae* (12%), *Staphylococcus* (11%), *Klebsiella* (7%), *Enterococcus* (5%), and *Prevotella* (4%) were the prevalent genera (Fig. 2).

Comparison of lung microbiome of COVID-19 with COVID-19 negative patients also demonstrated significant differences between the two groups by Similarity Percentage (SIMPER) analysis, which was used to define the OTUs that majorly contributed to the dissimilarity between the two groups. Specifically, the lung microbiome ecosystem of critically ill patients with COVID-19 was characterized by a higher relative abundance of *Pseudomonas* spp. (*p* value FDR-corrected = 0.021) compared to COVID-19-negative subjects (Fig. 2). On the other hand, the lower respiratory tract microbiome of COVID-19-negative patients was mainly characterized by the enrichment of *Haemophilus influenzae*, *Veillonella dispar*, *Granulicatella* spp., *Porphyromonas* spp., and *Streptococcus* spp. (*p* value FDR-corrected = 0.0001, 0.00015, 0.0015, 0.01, 0.012, respectively) (Fig. 2).

**Figure 3:**
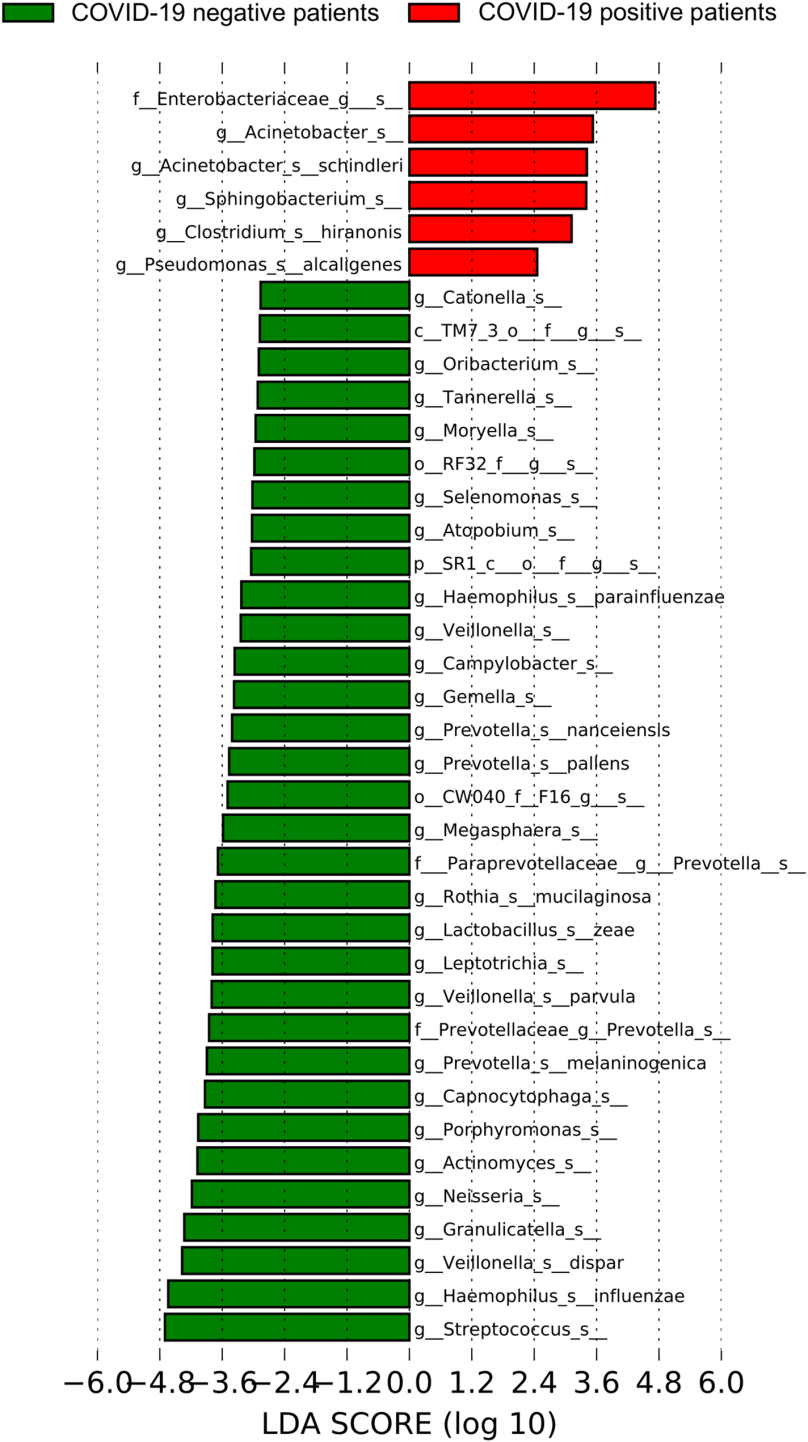
Plot from LDA LEfSE analysis. The plot was generated using the online Galaxy web platform tools at https://huttenhower.sph.harvard.edu/galaxy/. The length of the bar column represents the LDA score. The figure shows the microbial taxa with significant differences between the COVID-19 positive (red) and negative patients (green) (LDA score > 2) with their original identification codes.

The linear discriminant analysis (LDA) effect size (LEfSE) algorithm highlighted that lung microbiome of the critically ill patients with COVID-19 was characterized by the presence of *Pseudomonas alcaligenes*,* Sphingobacterium* spp.,* Clostridium hiranonis*,* Acinetobacter schindleri*, *Enterobacteriaceae* of unknown genus and *Acinetobacter* spp. (LDA score > 2) (Fig. 3). In particular, *Enterobacteriaceae* of unknown genus dominated lung microbiota of three out of 24 critically ill COVID-19 patients. In contrast, the taxa that characterized the low respiratory tract microbiome in the COVID-19 negative patients included, *Streptococcus* spp., *Haemophilus influenzae*, *Granulicatella* spp., *Veillonella dispar*, *Porphyromonas* spp., and *Neisseria* spp. (LDA score > 2) (Fig. 3). A complete display of the taxa which were identified as potential biomarkers of the lung microbiome of patients with COVID-19 and patients with pneumonia at LDA > 2 threshold are shown in Supplementary Fig. S1 in the Supplementary data.

In addition, we examined the association of antiviral and immunomodulatory treatments on the lung microbial community in COVID-19 infected critically ill patients, but we did not observe any differences in the lung microbiome according to antiviral or immunomodulatory treatments (data not shown).

### Lung bacterial relative abundance and culture isolation

Analysis of relative abundance (RA%) in relation to *P. aeruginosa* infection showed that 4 out of 24 (17%) COVID-19 patients developed lower respiratory infection (LRI), while only one (4.1%) COVID-19-negative patient developed LRI. Of note, all COVID-19 patients who developed LRI due to *P. aeruginosa* (n = 4, 100%) had a pulmonary RA% of *Pseudomonas* genus higher than 8%. Amongst the positive patients who did not develope infection due to *P. aeruginosa* (n = 20), 85% had a pulmonary RA% of *Pseudomonas* spp. lower than 8% (n = 17) and only 15% (n = 3) had a higher RA% (Supplementary Fig. S2, panel a).

Among critically ill patients included in this study, six patients with COVID-19 (25%) developed infection due to carbapenem-resistant *A. baumannii* (CR-Ab), 50% (12/24) of COVID-19 patients were colonized with CR-Ab, while only one (4%) COVID-19 negative patient was colonized with CR-Ab. Correlation of CR-Ab isolation with RA% showed that one out of three patients with RA% of *Acinetobacter* higher than 10% developed CR-ab infection (Supplementary Fig. S2, panel b).

In relation to *Enterobacteriaceae* infection, six out of seven (85%) COVID-19 patients with RA% higher than 8% for Enterobacteria had a pulmonary infection (Supplementary Fig. S2, panel c).

### Incidence of lower respiratory tract infections during COVID-19 epidemic

**Figure 4:**
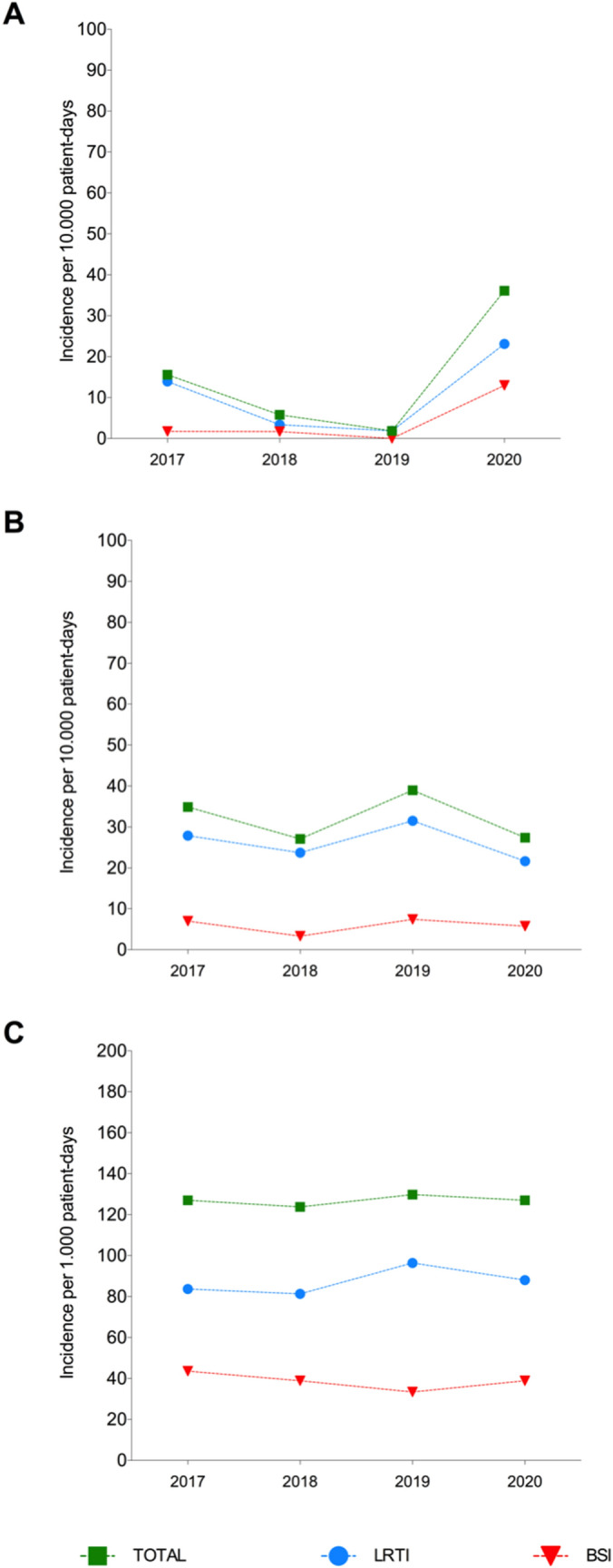
(**a**) Incidence rate of ICU-acquired infection due to Carbapenem-resistant *Acinetobacter baumannii* (CR-Ab) per 10.000 patient-days over the same 4-months period (January–April) from 2017 to 2020. (**b**) Incidence rate of ICU-acquired infection due to *Pseudomonas aeruginosa* and (**c**) carbapenemase-producing *Enterobacteriaceae* (CPE). Abbreviations: Lower Respiratory Tract Infection, LRTI; Bloodstream Infection (BSI).

We retrospectively analyzed microbiological data from critically ill patients recovered in ICUs over the same period (i.e. January–April) from 2017 to 2020 to compare the incidence of CR-Ab and *P. aeruginosa* infections in non-COVID versus COVID-19 patients. In the first 4-months of 2020, a total of 1317 patients were admitted to the ICUs with a mean length of stay of 5.3 days and a total of 6924 ICU patient days. Our results indicate that the incidence rate of ICU-acquired infection due to CR-Ab was significantly higher (incidence rate ratio 0.05; 95% CI 0.001–0.31, *p* value = 0.0001) during the first 4-months of 2020 in comparison to previous year (Fig. 4a). Also, significant increase in the incidence rate of CR-Ab infection between 2020 and 2018 or 2017 was observed with Poisson regression model (*p* value = 0.005). In particular, the relative risk of ICU-acquired BSI due to CR-Ab during the first 4-months of 2020 was 7.44 to 7.68-fold higher than previous years, while relative risk for LRI was 1.66 to 12.48-fold higher than three previous years.

In parallel, retrospective analysis of *P. aeruginosa* infection among critically ill patients showed no significant differences over the last 4 years. Specifically, the incidence rate of BSI and LRT due to *P. aeruginosa* ranged from 0.33–0.74 and 2.79–3.15 person-days, respectively from 2017 to 2020 (Fig. 4b). Similarly, there was no significant difference in the incidence of carbapenemase-producing *Enterobacteriaceae* over the 4-year observation period (Fig. 4c).

## Discussion

In this study, we characterized the microbial community of lower respiratory tract of critically ill COVID-19 patients in comparison with COVID-19-negative patients with pneumonia. Our results suggested that although microbial richness did not differ significantly between COVID-19 and non-COVID-19 infected patients, significant microbial diversity could be demonstrated in the lung microbiota between non-COVID-19 versus COVID-19 infected critically ill patients. The dysbiosis observed in critically ill patients with COVID-19 was characterized by reduction of commensal bacterial species and enrichment of opportunistic gram-negative pathogens frequently associated with multidrug resistance.

Several studies have reported that lung viral infections can drive change of the bacterial community by modifying both local microbial composition and total amount of bacterial load^20–22^. The composition of lung microbiota of healthy subjects is mainly characterized by commensal bacteria (*Prevotella* spp., *Veillonella* spp., *Streptococcus* spp. and *Tropheryma whipplei*) which are involved in the maintenance of the host immune homeostasis^20^. Recently, Shen and co-workers reported that lung microbial composition of SARS-CoV-2-infected patients was dominated by pathogens or elevated levels of commensal bacteria^23^. Although limited by the small sample size, the authors observed that the microbiome signatures from BAL samples collected from COVID-19 patients were similar to that of patients with community-acquired pneumonia and differed from that of healthy controls. At the same time, a recent study conducted at autopsy from 20 deceased COVID-19 patients demonstrated that lung microbial composition was dominated by complex mixed bacterial infections mainly characterized by *Acinetobacter*, *Chryseobacterium*, *Burkholderia*, and *Enterobacteriaceae*^7^. Our results are in agreement with these findings as we found that commensal bacteria including *Veilonella* spp. *Prevotella* spp. *Neisseria* spp. and *Streptococcus* spp. were diminished in lower respiratory tract of the COVID-19 patients, while lung microbiota of COVID-19 critically ill patients was characterized by opportunistic pathogens, including *Pseudomonas* spp., *Enterobacteriaceae* and *Acinetobacter* spp. Most of these pathogens represent the major causes of hospital-acquired infections, which are frequently associated with resistance to multiple antibiotic classes and high mortality rates^24^.

Several studies have reported that a variable percentage of COVID-19 patients (4–20%) have bacterial and/or fungal coinfection. In recent meta-analysis studies, bacterial co-infection was reported in the 7% of hospitalized patients with COVID-19 and up to 14% in critically ill patients or for secondary infection^4,25^. The most frequently reported bacteria in co-infected patients with COVID-19 included *P. aeruginosa*, *H. influenzae* and *Enterobacteriaceae*^5,25^. We recently reported on a clonal outbreak of carbapenem-resistant *A. baumannii* (CR-Ab) infections in COVID-19 ICUs^26^. Here we reported that CR-Ab infection increased during COVID-19 in comparison to previous years, while infections due to carbapenemase-producing Enterobacteria (CPE) and *P. aeruginosa* remained stable. Based on these findings, we hypothesized that dysbiosis with enrichment of gram-negative species observed in critically ill patients was mainly due to COVID-19 infection rather than mechanical ventilation. These results are in accordance with previous studies showing that coinfection rate was higher in severely affected patients with COVID-19 due to nosocomial multidrug-resistant pathogens (*A. baumannii*, *Escherichia coli*, *P. aeruginosa* and *Enterococcus*) especially among ICU death patients [unpublished data].

Our study is unique from previous lung microbiome investigations of critically ill COVID-19 patients in that we compared the microbial community of the lower respiratory tract from these patients to contemporary patients at the same hospital with non-COVID-19 pneumonia. However, this comparison group was not ideal as they were not as critically ill as the COVID-19 positive patients. This limitation reflects the reality of performing studies during the major pandemic wave of March–April 2020 when ICUs were overwhelmed with COVID-19 patients and few contemporary non-COVID-19 patients were available for analysis. Therefore, further prospective studies will be performed to compare critically ill patients. Another limitation is that lung microbiota was performed on small number of patients treated with several anti-viral and immunosuppressive treatments. Further prospective studies enrolling a larger number of participants will be necessary to evaluate the lung microbiome dysbiosis in relation to the different treatments. In addition, lower respiratory samples were collected at different time after symptoms onset (median 15, IQR 8.5–27). Although samples from COVID-19 patients were collected mainly at 7 days after ICUs admission, we hypothesize that temporal changes of lung microbial composition occurred during COVID-19 infection. Further study is necessary to confirm this hypothesis.

In summary, we found that the lower respiratory tract microbial community of critically ill patients with COVID-19 infection differs significantly from COVID-19-negative patients with pneumonia, and this difference characterized by predominance of gram-negative bacteria that are predisposed to multidrug resistance phenotypes. It has been hypothesized that several factors could contribute to the emergence of infections due to antimicrobial-resistance pathogens in critically ill patients including ICU stay, mechanical ventilation, high concentration of pro-inflammatory cytokines released (e.g. IL-2, IL-6, TNF–α), aggressive use of immunomodulatory therapies, and overuse of antibiotics^27,28^. Further larger studies should be performed to investigate whether specific anti-inflammatory and/or antiviral treatment could be associated to specific microbiome composition and predict predisposition of critically ill patients to secondary infection.

## Methods

### Study participants/design

From 1st April through May 31th 2020, a total of 48 patients recovered at the S. Orsola-Malpighi University-Hospital (Bologna, Italy) were included in the study. Study group consisted of 24 patients positive to SARS-CoV-2 by RT-PCR on nasopharyngeal swabs or lower respiratory tract samples and comparison group comprised 24 patients with pneumonia and negative to SARS-CoV-2. The study was conducted in accordance with the Declaration of Helsinki. Samples were coded and analysis was performed with anonymized database. Informed consent for study participation was obtained from each patient. The study was approved by the local IRB (Comitato Etico AVEC) with approval number n. 283/2020/Oss/AOUBo.

### Lower respiratory sample collections and microbiological analysis

Bronchoalveolar lavage (BAL) samples were collected by bronchoscopy with 5–30 mL of isotonic sterile solution. The BAL samples were collected between April 3th and May 5th 2020 from COVID-19 and negative patients recovered in tertiary teaching hospital (Policlinico S.Orsola-Malpighi) located in Bologna. Policlinic Sant’Orsola is a University Hospital with 1,420 beds and an average of 72,000 admissions per year. Each sample was cultured on selective agar plates for 48 h at 37 °C. Samples were considered significant by containing > 10^4^ CFU/mL. Isolates were identified by MALDI-TOF MS (Bruker, Germany) and antimicrobial susceptibility testing was performed by Microscan WalkAway system (Beckman Coulter, USA). MIC results were interpreted according to EUCAST clinical breakpoints (available at: https://eucast.org/clinical_breakpoints/).

### Microbiome sequencing

Total microbial DNA was extracted from samples using the QIAamp 96 PowerFecal QIAcube HT kit on the QIAcube HT instrument (QIAGEN, Hilden, Germany) following the manufacturer’s instructions. A bead‐beating step with Lysing Matrix E (MP Biomedicals) was performed on a FastPrep24 bead-beater (MP Biomedicals, Irvine, CA) at 6.0 movements per second for 40 s, before total DNA extraction. Negative controls of sequencing run were PCR-grade water which underwent library preparation steps and Next Generation Sequencing (NGS). DNA was quantified using the Qubit™ 4 Fluorometer (Fisher Scientific). V3 to V4 region of the 16S rRNA gene was amplified using the primer set S‐D‐Bact‐0341‐b‐S‐17/S‐D‐Bact‐0785‐a‐A‐21^8^. We tested these primers on the commercial mock community ZymoBIOMICS Microbial Community standard (Zymo Research) as a positive control to assess the performance of the DNA extraction and sequencing procedures. PCR products were purified with a magnetic bead‐based clean‐up system (Agencourt AMPure XP; Beckman Coulter, Brea, CA). Indexed libraries were prepared by limited‐cycle PCR using Nextera technology and further cleaned up with AMPure XP magnetic beads (Beckman Coulter). Libraries were pooled at equimolar concentrations (4 nM), denatured, and diluted to 5 pM before loading onto the MiSeq flow cell. Sequencing on Illumina MiSeq platform was performed by using a 2 × 250 bp paired end protocol, according to the manufacturer's instructions (Illumina, San Diego, CA).

### Data analysis

Paired-end sequenced reads of samples were analysed combining PANDAseq2 and the wrapper package Quantitative Insights Into Microbial Ecology (QIIME) version 1.9.1^9,10^. High‐quality reads were binned into operational taxonomic units (OTUs) at a 0.97 similarity threshold using UCLUST^11^. For bacterial taxonomy assignment, Greengenes database from May 2013 release (http://greengenes.secondgenome.com/downloads) was used. Chimera filtering was performed discarding singleton OTUs. Samples that had less than 1000 reads after Illumina MiSeq sequencing were discarded. The bacterial relative abundance data were imported into R (version 3.6.1) on Rstudio v1.1.456 where all statistical analysis was performed using R package *phyloseq*^12,13^. Taxa that were present in less than 2% of the samples and environmental microbial contaminants were excluded from the present analysis by filtering out OTUs that were specifically present in the negative controls (water) using the *decontam* R package at 1% stringency. The differences in alpha diversity were evaluated, based on the data distribution of metrics, using ANOVA and Tukey’s HSD (honestly significant difference) tests for normally distributed data or Wilcoxon–Mann–Whitney with Holm-Bonferroni correction method for non-normally distributed data. To compare microbial composition between samples, beta diversity was measured by calculating the weighted or unweighted UniFrac distance matrix. Principal coordinates analysis (PCoA) was applied on the distance matrices to generate bi-dimensional plots in R. Dispersion of the PCoA clusters was compared using the *betadisper* function in R *vegan* package^14^. If there were no significant differences in beta dispersion, the permutational analysis of variance (PERMANOVA) test, calculated using the function *adonis* in the *vegan* package^15,16^, was performed to determine whether there was a significant separation between different sample groups. The plots were graphed using *ggplot2* R packages^17^. Dissimilarity percentage (SIMPER) analysis function^18^ based on R packages *vegan* and *dplyr* (https://github.com/asteinberger9/seq_scripts) was used to determine the contribution of individual taxa driving the average dissimilarities between groups. A *p* value < 0.05 after False Discovery Rate (FDR) correction was considered as statistically significant. Linear discriminant analysis (LDA) effect size (LEfSE) algorithm^19^, a tool which is hosted on the Galaxy web application at https://huttenhower.sph.harvard.edu/galaxy/, was also used to discover potential bacterial biomarkers associated to COVID-19 patients. The differences in abundance were regarded as significant when the logarithmic LDA score was higher than 2.

### Clinical data and statistical analysis

The incidence rate was calculated as the number of BSIs due by CR-Ab, *Pseudomonas aeruginosa* and carbapenemase-producing *Enterobacteriaceae* (CPE) divided by the total number of 1000 person-days at risk in ICUs at the Policlinico S. Orsola-Malpighi in Bologna. The incidence rate was calculated for the first 4-months of 2020 (i.e. during COVID-19 pandemic) and for the last 3 years over the same 4-month period (i.e. January–April). Poisson regression was used to compare Incidence rate ratios (IRR) and 95% CI of infection rates due to *Acinetobacter*, *Enterobacteriaceae* and *Pseudomonas* for 2020 in comparison to the three previous years.

## Supplementary Information

Supplementary Information.

## Data Availability

The sequencing reads generated during the current study are available via the NIH Sequence Read Archive (SRA) via Bioproject PRJNA687143.
