# Supplementary material for: The lower respiratory tract microbiome of critically ill patients with COVID-19
Source: Sci Rep. 2021 May 12;11:10103. doi: 10.1038/s41598-021-89516-6 (PMC8115177; doi:10.1038/s41598-021-89516-6)
Supplement: Supplementary file 1 — Supplementary Information. [file 41598_2021_89516_MOESM1_ESM.docx]

**The lower respiratory tract microbiome of critically ill patients with COVID-19.**

Paolo Gaibani^1^, Elisa Viciani^2^, Michele Bartoletti^3^, Russell E. Lewis^3^, Tommaso Tonetti^4^, Donatella Lombardo^1^, Andrea Castagnetti^2^, Federica Bovo^1^, Clara Solera Horna^3^, Marco Ranieri^4^, Pierluigi Viale^3^, Maria Carla Re^1^, and Simone Ambretti^1^

^1^ Operative Unit of Clinical Microbiology, S. Orsola-Malpighi University Hospital IRCCS, Bologna, Italy

^2^ Wellmicro s.r.l, Via Piero Gobetti, 101, 40129 Bologna, Italy

^3^ Alma Mater Studiorum – Università di Bologna, Dipartimento di Scienze Mediche e Chirurgiche, Operative Unit of Infectious Diseases. S. Orsola-Malpighi University Hospital IRCCS, Bologna, Italy

^4^ Alma Mater Studiorum – Università di Bologna, Dipartimento di Scienze Mediche e Chirurgiche, Anesthesia and Intensive Care Medicine IRCCS, Policlinico di Sant’Orsola, Bologna, Italy

**Supplementary Fig. S1**. Dot plots of OTUs defined as significantly differentially abundant between the COVID-19 positive (blue) and negative patients (red) (LDA score > 2) with their original identification codes.


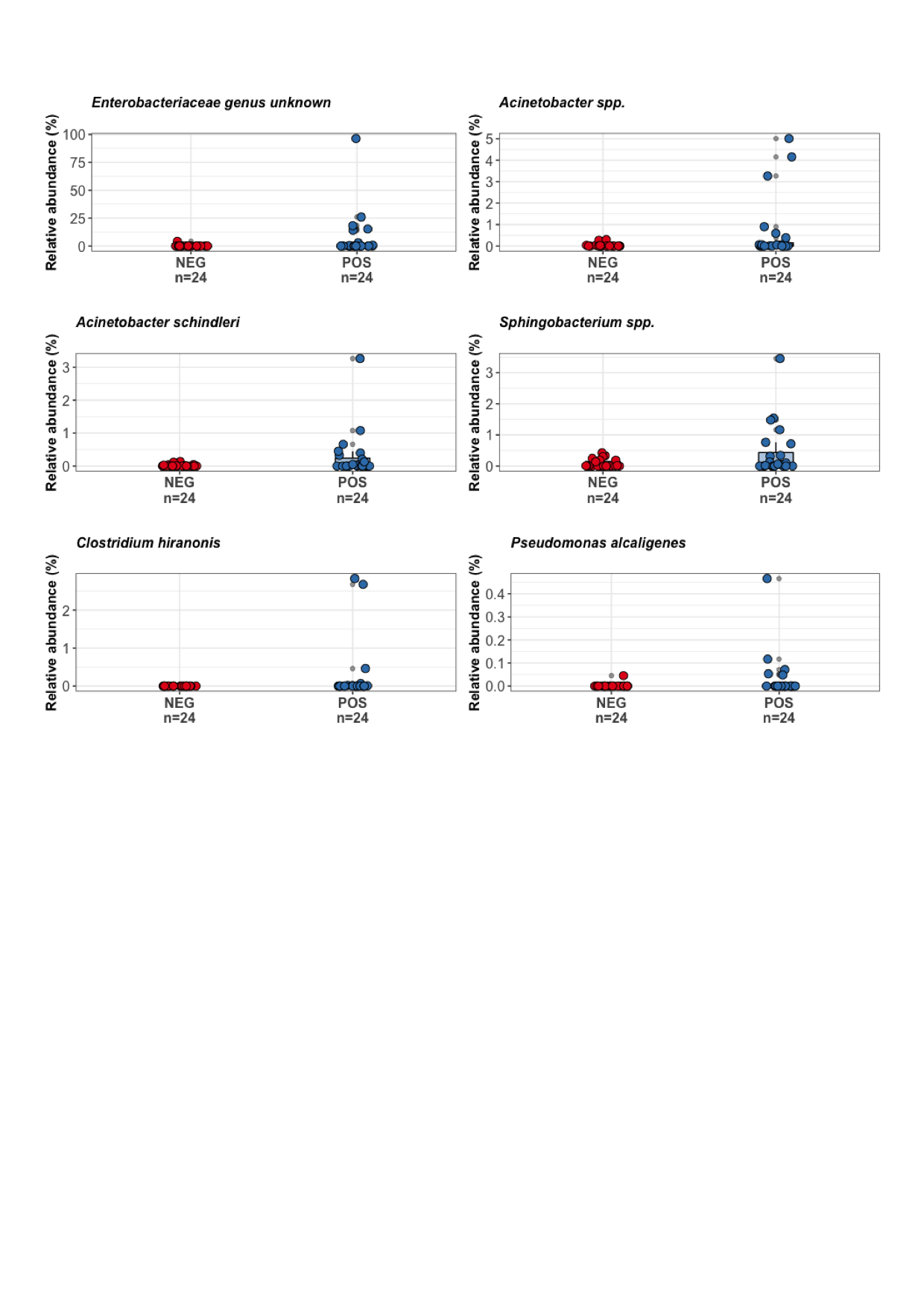


**Supplementary Fig. S2.** Bar plots of *Moraxellaceae* (**a**) and *Pseudomonadaceae* (**b**), and *Enterobacteriaceae* family (**c**) showing their Relative Abundances (RA%) in BAL from every patient and the presence (red bars) or absence (blue bars) of concomitant reported clinical infection.


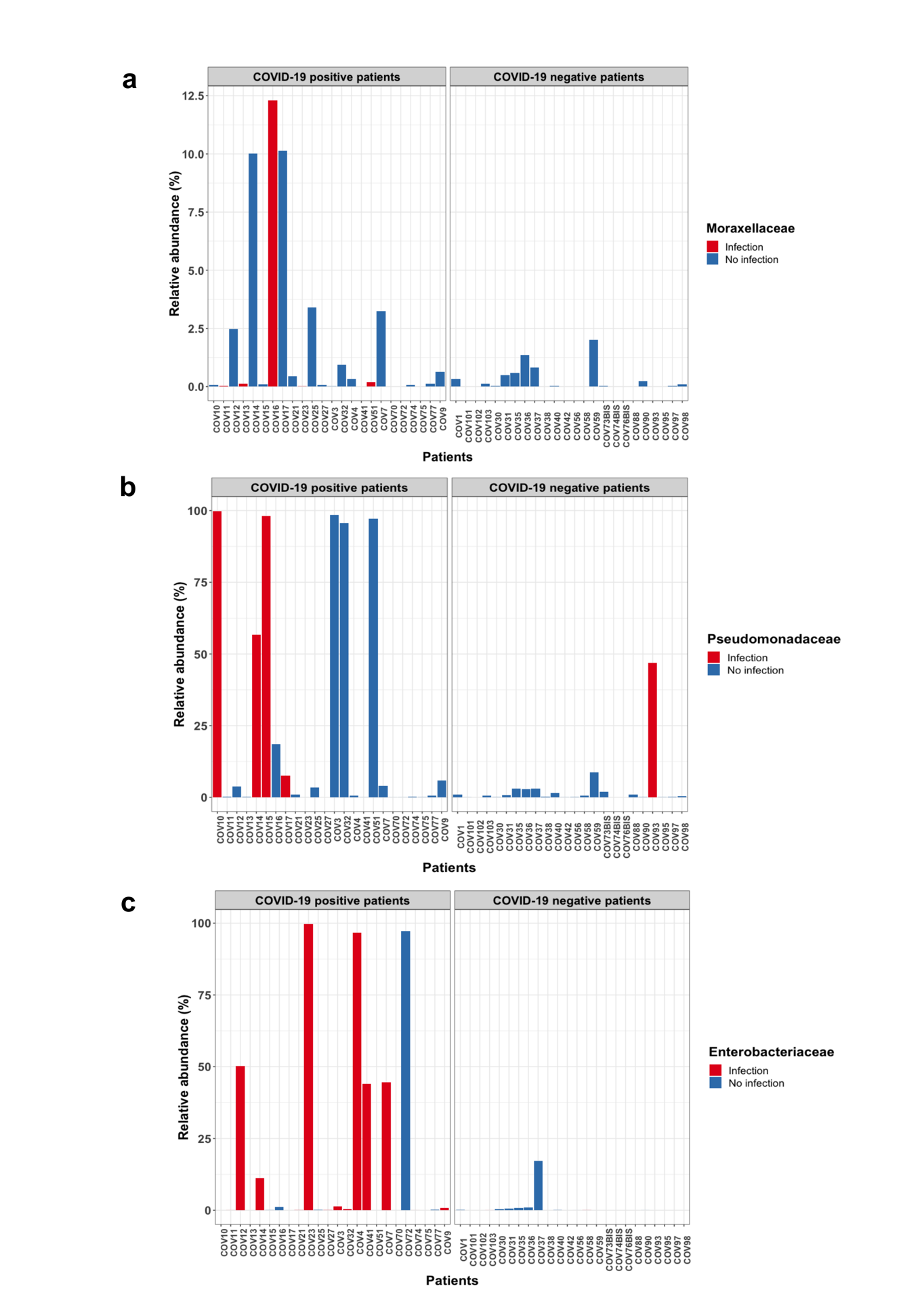


**Supplementary Fig. S3.** Dot plots of bronchoalveolar lavages (BAL) microbiome of COVID-19 positive and negative patients. The plot shows the sample distribution relative to the number of observed species and sequence reads per sample. The color of each point ranges from light yellow to black based on the value of the Shannon index calculated for each sample. The BAL samples present with a high alpha diversity at high as well as at low (above 1,000) sequence reads per sample.

**
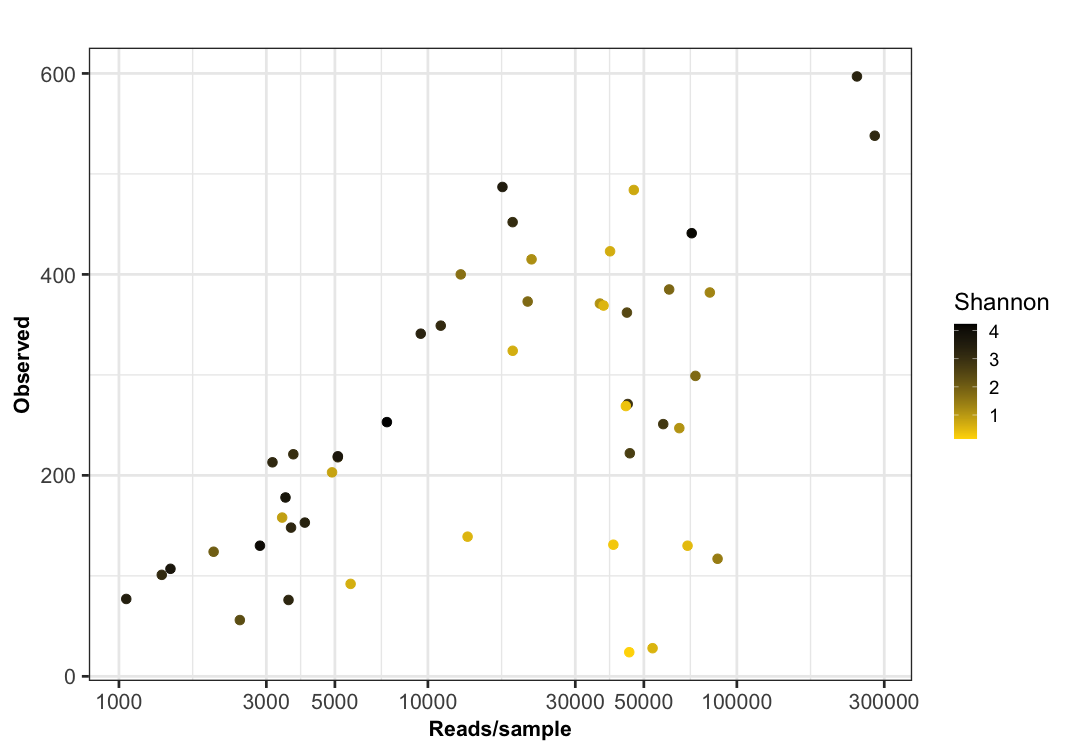
**
